# Spatiotemporal Distribution, Risk, and Source Identification of Organochlorine Pesticides in the Surface Water of Shitalakshya River, Bangladesh

**DOI:** 10.1155/jt/9611223

**Published:** 2026-08-04

**Authors:** Md. Mazharul Islam, Mohammad Shoeb, Md. Iqbal Rouf Mamun

**Affiliations:** ^1^ Department of Chemistry, University of Dhaka, Dhaka 1000, Bangladesh, du.ac.bd

**Keywords:** organochlorine pesticides, risk profile, Shitalakshya river, source identification, spatiotemporal dynamics

## Abstract

This study investigates the spatiotemporal distribution of organochlorine pesticides (OCPs) in the surface water (*n* = 60) of Shitalakshya River, Bangladesh, by an optimized and validated LLE–GC–ECD method by calibration (0.10–5.00 µgL^−1^), linearity (*R* ^2^ = 0.995–0.999), recovery (80.11%–117.10%), precision, specificity, selectivity, LODs (0.0009–0.0026 µgL^−1^), and LOQs (0.0027–0.0079 µgL^−1^). Among the targeted OCPs, α‐BHC (0.01–0.19 µgL^−1^), δ‐BHC (0.01–0.27 µgL^−1^), chlordane (0.01–0.23 µgL^−1^), endosulfan (0.01 and 1.56 µgL^−1^), 4,4′‐DDE (0.01–2.14 µgL^−1^), dieldrin (0.03–0.58 µgL^−1^), endrin (0.01–2.96 µgL^−1^), 4,4′‐DDD (0.01–4.07 µgL^−1^), endrin aldehyde (0.01–3.72 µgL^−1^), 4,4′‐DDT (0.01–0.02 µgL^−1^), and endosulfan sulfate (0.03–2.05 µgL^−1^) were detected with varying frequencies. These levels were below the international guideline values, except 4,4′‐DDE in Kanchon (2.14 μgL^−1^), dieldrin in Kanchpur (0.10 μgL^−1^), Atlapur (0.12 μgL^−1^), Ghorashal (0.45 μgL^−1^), Charshindur (0.58 μgL^−1^) and Kapasia (0.03 μgL^−1^), endrin in Kapasia (2.96 μgL^−1^), 4,4′‐DDD in Kalagachhia (1.63 μgL^−1^), Narayanganj (0.69 μgL^−1^), Nabiganj (1.95 μgL^−1^), Ichakhali (1.05 μgL^−1^), Kanchon (0.57 μgL^−1^), Atlapur (1.02 μgL^−1^), Ghorashal (2.51 μgL^−1^), and Charshindur (4.07 μgL^−1^). The occurrence of OCPs exhibited pronounced spatiotemporal variation, with the highest concentrations and detection frequencies observed during the dry seasons (L. autumn, autumn, winter, and spring), indicating reduced dilution and possible remobilization from contaminated sediments. Multivariate statistical analysis revealed distinct sources, with endosulfans and BHCs dominating at Kanchon, whereas dieldrin, endrin, and chlordane were more strongly associated with Ghorashal and nearby locations, suggesting localized anthropogenic inputs and legacy pollution sources. While risk is generally low, specific exceedances (*H*_*q*_ > 1) were observed in L. Autumn 2022 for 4,4′‐DDD, indicating a localized noncarcinogenic hazard. While the immediate screening‐level risks are low across most seasons, the high chemical persistence and bioaccumulation over time warrant continued monitoring and proactive water management.

## 1. Introduction

Chemical agents known as pesticides are used to manage pests that pose a risk to property, public health, and agriculture [1]. Because of high lipophilicity and strong environmental persistence, organochlorine pesticides (OCPs) are a historically significant subgroup [2]. OCPs like DDT, HCH, aldrin, dieldrin, and chlordane, which were widely used in the middle of the 20^th^ century, transformed agriculture and public health by raising crop yields and lowering vector‐borne illnesses like typhus and malaria [3, 4]. Governments and aid schemes boosted their widespread application, which led to usage ranging from grain protection to termite management. However, OCPs’ effectiveness was accompanied by severe harmful effects on human health and other animals in the environment. Global apprehension was stirred up by their toxicity and persistence, which were strikingly exhibited in Silent Spring (1962) [5]. As a result, the Stockholm Convention instituted international directives and broad prohibitions [6]. OCP residues are still found in environmental compartments such as soil, water, and other biological indicators despite restrictions, demanding ongoing environmental monitoring. OCPs can undergo long‐range atmospheric transport (LRAT) through air and water due to their semivolatile nature and chemical stability, which permit them to endure for decades in air, soil, sediment, and aquatic bodies [7]. Lipophilic properties stimulate trophic biomagnification and bioaccumulation in the food chain, leading to elevated concentrations in fish, birds, and human bodies [8, 9]. These compounds cause population decline and ecological insecurity by meddling with reproduction, altering the function of developmental systems, and disrupting endocrine systems in aquatic creatures [10, 11]. Human exposure to OCPs occurs via ingestion of contaminated food, water, air, and skin, and even trace levels can cause immunological disease, cancer, neurotoxicity, hepatotoxicity, and reproductive dysfunction [12–14]. In order to protect ecosystems and community health, strict laws and regulations, risk estimation, and sustainable eco‐friendly alternatives are obligatory, as highlighted by the environmental impact of OCPs. Because of decades of mechanized agricultural production and negligent supervision of regulations, OCPs continue to pose a persistent environmental hazard in South Asia. OCPs like DDT, HCH, aldrin, and endosulfan have long been used by countries like India, Bangladesh, Pakistan, and Nepal to amplify crop yields and manage vector‐borne disease [15, 16]. Despite the fact that many OCPs are currently banned worldwide, hidden illegal use, leakage from antiquated stockpiles, and long‐range transboundary conveyance are the main factors for their existence [6]. The issue is exacerbated by regional climatic factors; monsoon‐driven effluent, dense agricultural production, and low‐grade waste management allow OCPs to enter rivers, wetlands, and coastal sites. Consequently, major river systems, i.e., the Ganges, Brahmaputra, and Indus, serve as sinks for pesticides, threatening ecosystem health, food security, and fisheries [17]. In the past, Bangladesh has widely used OCPs, especially DDT and lindane, due to climate conditions that are beneficial to pest growth [18, 19]. Historically, Bangladesh operated a state‐owned DDT production factory in Chittagong, which was closed in the early 1990s [20, 21], alongside widespread technical‐grade HCH and DDT applications in regional agriculture and public health campaigns. This regional industrial and agricultural legacy can be reasonably linked to current water contamination profiles; due to the high environmental persistence and high octanol‐water partition coefficients (*K*_ow_) of these legacy chemicals, massive historical loads remain bound to soil matrices within the catchment basin. Consequently, evaluating the Shitalakshya River water column provides a direct window into how these regional historical usage footprints are continuously mobilized into active aquatic ecosystems via modern catchment dynamics. In Bangladesh, human exposure mostly occurs from drinking polluted water and consuming fish, but a thorough grasp of pollution and related hazards is constrained by a lack of monitoring and proper datasets.

Since the Shitalakshya River is one of Bangladesh’s most severely affected river systems, it was chosen for this study due to its scientific, environmental, and global significance. OCPs have a greater chance of spreading downstream and across borders due to the Shitalakshya’s hydrological connections to the Meghna River system and, eventually, the Bay of Bengal. Because of this, the river serves as an essential natural laboratory for researching the movement and fate of persistent organic pollutants (POPs) in tropical river basins that are influenced by the monsoon. While historical investigations have broadly documented the presence of OCPs in various Bangladeshi river systems, existing literature predominantly relies on isolated, single‐season screenings or focuses exclusively on total concentrations. Significant knowledge gaps persist regarding how specific legacy chemical profiles dynamically shift across high‐resolution seasonal milestones such as the transition from high‐dilution monsoon phases to low‐flow dry periods. Furthermore, localized multipathway risk calculations for distinct age cohorts under realistic chemical mixture exposure remain severely underdeveloped in regional frameworks. This study addresses these gaps by providing a systematic, six‐season spatiotemporal assessment of 20 OCPs at ten stations in the Shitalakshya River by using an optimized and validated LLE–GC–ECD protocol, shifting the paradigm from broad regional surveys to compound‐specific mixture toxicity and diagnostic‐ratio mapping. We hypothesize that total OCP concentrations will peak significantly during low‐flow dry seasons (L. autumn and winter) due to reduced river dilution capacity, whereas monsoon periods will exhibit a marked dilution effect. Spatially, we expect contamination profiles to diverge sharply by station type, with heavily industrial–urban junctions like Kanchon and Ghorashal displaying elevated concentrations compared to agricultural reference points due to proximity to dense historical catchments. Finally, we hypothesize that diagnostic ratios for DDT and HCH classes will uniformly fall below 0.5, suggesting that the contamination is entirely driven by weathered historical residues rather than active modern inputs.

## 2. Materials and Methods

### 2.1. Sampling Area

**FIGURE 1:**
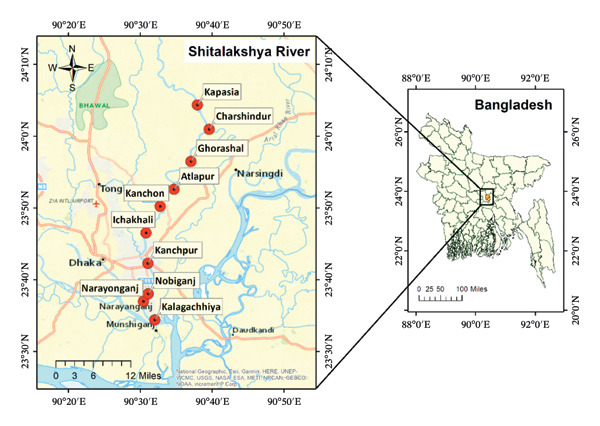
Sampling stations in Shitalakshya River for the analysis of OCPs.

Dhaka, the capital and fastest growing megacity of Bangladesh, is located north of the Buriganga River and covers 306.38 square kilometers between 23.69°–23.89° N and 90.33°–90.44° E [22, 23]. The population density of urban areas ranges from 30,000 to 40,000 people/km^2^, whereas that in rural areas ranges from 2900 to 3900 people/km^2^. As a developing country, Bangladesh faces challenges like improper management and unplanned urbanization, leading to environmental degradation [24]. Dhaka is connected to six rivers, including the Turag, Dhaleshwari, Shitalakshya, Buriganga, Balu, Meghna, and the Tongi Khal canal [25]. The Shitalakshya River, 110 km long, with an average depth of 10 m, flows through both urban and rural areas, remaining navigable year‐round. Its mean annual flow reaches 74 m^3^/s measured at Demra, with the maximum depth at 21 m and its widest site, near Narayanganj, at 300 m [26]. Figure 1 represents the position of the sampling stations along the Shitalakshya River, Bangladesh, selected for OCP analysis. Sampling stations were strategically selected to represent diverse anthropogenic activities, land‐use patterns, and hydrological conditions along the river. Stations targeted potential key pollution hotspots, including industrial, agricultural, textile, and municipal influence zones, as well as areas of public health significance and cumulative downstream exposure. This upstream‐to‐downstream design enabled assessment of localized pollution inputs, diffuse contamination sources, and overall contamination gradients within the river system.

The Shitalakshya River basin is situated within the Bengal Basin and is underlain by Quaternary alluvial deposits consisting of sand, silt, and clay, which influence its hydrogeochemical characteristics. The river is sustained by rainfall and upstream tributaries, exhibiting pronounced seasonal fluctuations driven by monsoonal recharge and dry‐season water depletion. Water quality is strongly affected by anthropogenic activities, including urbanization, industrialization, and agriculture. Urban stations such as Kalagachhia, Narayanganj, Nabiganj, Kanchpur, Ichakhali, and Kanchon are primarily influenced by industrial effluents, municipal wastewater, domestic sewage, and urban runoff, whereas the rural stations (Atlapur, Ghorashal, Charshindur, and Kapasia) are predominantly impacted by agricultural runoff, rural domestic wastewater, and natural organic inputs. The diversity of pollution sources underscores the combined influence of probable industrial, urban, and agricultural activities on the environmental quality of the Shitalakshya River.

The sampling stations of the Shitalakshya River for the analysis of OCPs are reported in Table 1. The table summarizes the geographic coordinates, sampling time span, land‐use characteristics, and stream direction of each location, ensuring precise spatial identification and reproducibility of sampling [27]. The sampling period was categorized into six seasons according to the climatic conditions of Bangladesh: autumn 2022 (September–October 2022), L. autumn 2022 (November–December 2022), winter 2023 (January–February 2023), spring 2023 (March–April 2023), summer 2023 (May–June 2023), and rainy 2023 (July–September 2023). These seasonal designations were used consistently throughout the study for spatiotemporal analysis.

**TABLE 1:** The sampling stations of Shitalakshya River for the analysis of OCPs.

Sampling stations	Sampling duration	Latitude	Longitude	Area	Stream
Kalagachhia	Autumn 2022 (September 2022) to rainy 2023 (August 2023)	23.572918	90.533929	Urban	Downstream
Narayanganj		23.615840	90.507787	Urban	
Nabiganj		23.633149	90.518537	Urban	
Kanchpur		23.704235	90.518040	Urban	
Ichakhali		23.774942	90.513926	Urban	
Kanchon		23.836672	90.546451	Urban	
Atlapur		23.875924	90.578627	Rural	Upstream
Ghorashal		23.940812	90.617865	Rural	
Charshindur		24.015453	90.660157	Rural	
Kapasia		24.071971	90.632971	Rural	

The Shitalakshya River catchment is a highly dynamic socioecological system influenced by both intensive agricultural activities and rapid industrialization. Agricultural areas within the basin have historically utilized OCPs for crop protection, and residual pesticide deposits in soils continue to be transported into the river through irrigation return flows, rainfall runoff, and monsoon‐driven flushing. In contrast, urban and industrial sectors contain major facilities, including fertilizer industries, power plants, textile and dyeing factories, and paper mills, which contribute industrial effluents and municipal wastewater. Although these industries are not direct sources of OCP production, their discharges can alter water chemistry and influence the transport, partitioning, and remobilization of persistent pesticide residues. The contrasting land‐use patterns and pollution sources create distinct spatial variations in contaminant distribution throughout the river system.

### 2.2. Sampling Strategy, Pretreatment, and Preservation

A total of 60 water samples were collected across 10 stations over six seasonal campaigns (*n* = 10 per campaign). To maximize the spatial and temporal representation of the Shitalakshya River, a single grab sample was collected at each station per campaign, following established protocols for river monitoring. Samples were collected at a depth of 10–20 cm, maintaining a distance of at least 15–20 m from the shoreline to avoid localized boundary layer effects. This systematic sampling framework was specifically designed to guarantee statistical adequacy and capture both spatial and temporal environmental heterogeneities. Spatially, the 10 stations create a distinct gradient spanning 110 km, purposefully dividing the river into a rural upstream zone (4 stations) affected primarily by diffuse agricultural activities, and an urban downstream cluster (6 stations) directly exposed to concentrated municipal sewage and point‐source industrial effluents. Temporally, sampling was executed at fixed 2‐month intervals to capture the profound hydrological fluctuations driven by the local monsoonal climate. One dedicated sample was collected per station during each of these six distinct campaigns. This bimonthly pacing ensures representation across the extreme dry season (characterized by low dilution and high chemical concentration effects) and the heavy monsoon rainy season (characterized by intensive surface runoff from surrounding agricultural catchment areas), providing a statistically robust matrix for spatiotemporal characterization. The bottles (HDPE amber plastic bottles obtained from Thermo Scientific, USA) were prepared by rinsing them with detergents and thoroughly washing them multiple times using distilled water. Collected samples were immediately stored in insulated ice boxes containing blue ice packs to maintain a transportation temperature below 4°C. Upon arrival at the laboratory, all samples were stored in the dark at 4°C, and the holding times prior to extraction were strictly restricted to under 48 h.

### 2.3. Equipment and Chemicals for the Analysis of OCPs

The equipment used was GC–ECD (Model GC‐2030, Shimadzu, Japan), rotary vacuum evaporator (Heidolph, Germany), centrifuge machine (Sigma, Model‐2‐16 P, Heraeus Sepatech Labofuge A), separatory funnel (1000 mL), conical flask, round bottomed flask (250 mL), graduated pipette (10.0 and 5.0 mL), volumetric flasks (250 and 50 mL), Teflon tube, reagent bottles (Pyrex glass, England), funnels and GC vials, round bottom flask, and Pasteur pipette. The chemicals were *n*‐hexane (Daejung Chemicals, South Korea), DCM, concentrated H_2_SO_4_ (RCI Labscan Limited, Thailand), NaCl (Sigma‐Aldrich, Germany), anhydrous Na_2_SO_4_ (Scharlab S.L. Spain), and deionized distilled water. A standard mixture of 20 OCPs, such as α‐BHC, γ‐BHC, β‐BHC, δ‐BHC, heptachlor, aldrin, heptachlor epoxide, trans‐chlordane, cis‐chlordane, endosulfan I, 4,4′‐DDE, dieldrin, endrin, 4,4′‐DDD, endosulfan II, endrin aldehyde, 4,4′‐DDT, endosulfan sulfate, methoxychlor, and endrin ketone, with purities ranging from 96%–99% and a concentration of 2000 µgL^−1^ in a hexane/toluene (50:50) solvent, was purchased from Restek Corporation (Bellefonte, PA 16823, USA). The quantity of standard was 1 mL/ampule and stored in a freezer at < 10°C until analysis.

### 2.4. Sample Extraction for the Analysis of OCPs

Water samples were extracted following a modified USEPA (1993) protocol [28]. Initially, 500 mL of water sample was taken into a 1000‐mL separatory funnel, and the pH was corrected to 5.0 to 9.0 using NaOH or H_2_SO_4_. Dichloromethane (DCM, 30 mL) and a saturated solution of NaCl (25 mL) were added to it. The content was shaken vigorously for 10 min with occasional venting. After settling, the organic layer was isolated and collected in a 250‐mL Erlenmeyer flask. This extraction procedure was repeated twice with additional DCM [29, 30]. The extracts were combined and filtered via anhydrous Na_2_SO_4_, evaporated to complete dryness at 40°C, and dissolved again in 1.5 mL *n*‐hexane. An additional purification involved adding 1.5 mL *n*‐hexane (saturated with concentrated H_2_SO_4_), inverting the test tube for 30 s, and centrifuging for 5 min to separate the layers. The upper organic layer underwent silica gel column cleanup (*n*‐hexane: H_2_SO_4_, 2:1 g/g), passing through 6 mL *n*‐hexane for elution. Finally, the extract was concentrated to 1.5 mL under N_2_ and injected into the GC for the analysis of targeted OCPs. This multiphase procedure ensured effective isolation, purification, and concentration of analytes for reliable gas chromatographic detection and quantification [31].

### 2.5. Instrumental Conditions for the Analysis of OCPs

GC–ECD analytical conditions for the analysis of OCPs in river water are reported in Table 2. Analyses were completed using a 30 m Rtx‐CLPesticides2 column (0.32 mm i.d.) with a 1‐μL injection in splitless mode. The operational temperatures for injector and detector were set at 200°C and 300°C, respectively. N_2_ gas was used as carrier gas and make‐up gas (20 mL·min^−1^) [32]. The oven‐temperature program was from 150°C to 295°C, with a total run time of 35 min. Matrix‐matched standards and regular matrix spike recovery trials were processed alongside every batch. This allowed us to monitor, map, and mathematically subtract shifting baseline matrices and coeluting interferences. To calibrate the instrument, working standards for 20 OCPs across 0.10–5.00 μg·L^−1^ were prepared. The calibration curves demonstrated linearity with correlation coefficients (*R* ^2^ ≥ 0.990) within the acceptable range [33]. We confirmed analyte identity by comparing the retention times (RTs) of sample extracts with those of their corresponding standards.

**TABLE 2:** GC–ECD analytical conditions for the analysis of OCPs in river water.

Parameter	Conditions
Injector temperatures	200°C
Injection volume	1 μL
Injection mode	Split/splitless (splitless 1 min)
Column length	30 m
Column	Rtx‐CLPesticides2 columns
Column internal diameter	0.32 mm
Column oven	150°C (2 min hold), increase 5°C/min up to 295°C (4 min hold)
Detector temperature	300°C
ECD gas flow	20 mL/min.
Carrier and make‐up gas	N_2_
Run time (RT)	35 min.

### 2.6. Preparation of Standard Solutions of OCPs

An appropriate amount of standard reference sample was diluted to prepare a primary standard solution of 500 µgL^−1^. The solution was labeled indicating name and concentration and stored in the freezer (−20°C) for further use. The primary standard solutions were taken out of the freezer to equilibrate to room temperature. These primary standard solutions were diluted with *n*‐hexane to 200 µgL^−1^ as the middle standard solution. Then, working standard solutions of OCPs (0.10, 0.20, 0.50, 1.00, 2.00, and 5.00 µgL^−1^) were prepared by serial dilution with *n*‐hexane and transferred into 1.5‐mL GC vials and run on the GC–ECD by maintaining the optimized standard instrumental conditions.

### 2.7. Method Validation for the Determination of OCPs

The method was validated in terms of parameters such as calibration, matrix‐matched calibration, linearity, accuracy, precision, specificity, selectivity, LODs, and LOQs for the determination of OCPs in the surface water of the Shitalakshya River [32]. Matrix‐matched calibration curves were made through the fortification of OCPs working standards with the matrix (water). Then, homogenized by vigorous shaking and allowed to stand for 30–40 min before extraction to ensure the complete mixing of pesticides with matrix. The samples were then made ready by following the same extraction method as described before and making the final concentration 0.10, 0.20, 0.50, 1.00, 2.000, and 5.00 μgL^−1^. LOD and LOQ were calculated from the calibration line at low concentrations based on the standard deviation of the responses and slope [34, 35] by the following equations:

(1) LOD=3.3×standard deviation of the responseSlope of calibration curve,

(2) LOQ=10×standard deviation of the responseSlope of calibration curve.

The standard deviation of the response was obtained by the standard deviation of the *y*‐intercept of the regression line (*y* = mx + *c*) as it is assumed that the instrumental response (*y*) is linearly correlated with the concentration of *x* for a limited range of concentration [31]. The accuracy of the method was validated by calculating recovery percentages of spiked OCP standards (1.00 and 2.00 μg·L^−1^) in separate sample matrices. Each concentration was spiked in triplicate, homogenized, and left to stand for 30–40 min before extraction. The prepared samples were analyzed by GC–ECD, and recoveries were determined using the following equation:

(3) Recovery %=Amount foundAmount added×100.

To ensure data quality, all analytical procedures followed strict QA/QC protocols. This includes procedural blanks, laboratory blanks, and field blanks to monitor potential contamination, matrix spikes to assess recovery efficiency, and duplicate samples to evaluate analytical precision. All glassware was thoroughly cleaned, solvent‐rinsed, and baked prior to use, and high‐purity solvents and reagents were employed. No target OCPs were detected in the blank matrices above the LODs. Instrument performance was routinely verified through calibration checks, and quantification was based on matrix‐matched calibration standards. These measures ensured the accuracy and reliability of the analytical results. We acknowledge that surrogate standards and internal standards were not included in the analytical protocol. However, procedural blanks, laboratory blanks, field blanks, recovery studies, calibration verification, and method validation parameters were employed to ensure data quality.

### 2.8. Health Risk Assessment due to the Exposure of OCPs

The human health risk metrics—hazard quotient (*H*_*q*_), lifetime average daily dose (R), and cancer risk (*C*_*r*_)—rely on exposure parameters strategically chosen to model the demographics of the local riverine population (Table 3). Compounds with an *H*_*q*_ value greater than 1.0 may endanger ecosystems or have negative consequences. An *H*_*q*_ score below 1.0 denotes a comparatively minimal danger. An elevated ecological risk is correlated with a higher *H*_*q*_ value. Age‐stratified clusters were established to capture the heightened vulnerability of children and adolescents compared to adults, as physiological differences strongly influence chemical toxicity thresholds [37, 38]. The daily water ingestion metrics (C) utilize localized, age‐appropriate consumption baselines to prevent risk underestimation [37, 38]. The maximum possible lifetime cancer risk is when P exceeds 10^−4^, whereas *R* over 10^−6^ implies the highest risk of cancer. *R* at 10^−3^ suggests the necessity for safety precautions. For the cancer risk assessment (*C*_*r*_), global reference parameters were purposefully retained to ensure mathematical consistency with the integrated regulatory cancer slope factor (K) established by international databases [37, 38]. Similar to this, *C*_*r*_ > 10^−6^ points to the highest risk of cancer, while values *C*_*r*_ < 10^−3^ suggest the need for precautionary measures [37, 38].

**TABLE 3:** The established equations for the human health risk assessment of OCPs.

Risk assessment parameters	Term explanations
*H*_*q*_ = P/Q (no unit)	*H*_*q*_ = Hazard quotient, P = intake exposure level (mgkg^−1^day^−1^), *Q* = reference dose, which is to be consistent (2 × 10^−5^ mgkg^−1^day^−1^) [36]. The cumulative hazard index (HI) is the sum of individual *H*_*q*_.
*P* = (*A* × *B* × *C* × *D* × *E*)/(*F* × *G*) (mgkg^−1^day^−1^)	*A* = Average amount of OCPs found (mgL^−1^), *B* = fraction ingested (an absolute figure with a range of 0–1, however earlier research suggested *B* = 0.98), *C* = standard daily water intake (age‐based daily water consumption is as follows: 0–6 years = 0.3 L/day, 7–17 years = 1 L/day, and adults = 1.4 L/day) recommended by recent literature [37, 38]. *D* = exposure, frequency = 365 days/year, *E* = age‐based exposure duration: 0–6 years = 6, 7–17 years = 11, and adults = 35, *F* = average body weight; 15 kg for ages 0–6; 46 kg for ages 7–17; and 70 kg for adult, *G* = 2190 days for 0–6 years, 4015 days for 7–17 years, and 10,950 days for adults. The parameters are recommended by recent literature [37, 38].
*R* = (*A* × *B* × *C* × *D* × *E*)/(*F* × *G*) (mgkg^−1^day^−1^)	*R* = life average daily dose (mg/kg/bodyweight); for *R*, *G* = 70 years × 365 = 25,550 days, which is the same for all age groups [37, 38].
*C*_*r*_ = (*A* × *J* × *E* × *K* × *L*)/(*F* × *G*)	*C*_*r*_ = cancer risk, *J* = daily input (Lday^−1^); 2 Lday^−1^, *E* = 35 years, *F* = 60 kg, *G* = average life (year): 70 years × 365 = 25,550 days (for all age group); *K* = cancer slope factor (mgkg^−1^day^−1^): 0.07 (mgkg^−1^day^−1^); *L* = conversion factor: 10^−6^ [37, 38]. The total cancer risk (Ʃ*C*_*r*_) is the sum of individual cancer risks (*C*_*r*_).

Relying on these deterministic assumptions introduces clear margins of uncertainty. For example, applying a rigid ingestion fraction (*B* = 0.98) assumes that nearly all consumed water originates directly from the contaminated river source without alternative purification, which acts as a highly conservative, worst‐case exposure scenario. Furthermore, assuming a static adult exposure duration of 35 years or a fixed lifetime of 70 years (25,550 days) fails to capture the true variance in modern life expectancies and human migration behaviors. While these standardized boundaries are crucial for executing a screening‐level health risk matrix, they should be interpreted as conservative protective thresholds rather than precise individual medical forecasts.

## 3. Results and Discussion

### 3.1. Method Validation for the Determination of OCPs

Method validation parameters for the determination of OCPs in Shitalakshya River are reported in Table 4. Matrix‐matched calibration authenticates an interrelation between the concentration of OCPs and peak area, assuring precise quantification in sample matrices. The regression equations obtained from the calibration curves establish a consistent detector response for the targeted OCPs. The RTs (14.11–30.34 min) confirm actual chromatographic separation beyond coelution [39, 40]. Calibration curves showed strong linearity across the concentration range, with correlation coefficients (*R* ^2^) 0.995 to 0.999. This shows excellent performance according to the international acceptance benchmark regulation [41]. The sensitivity of the method was computed by LODs and LOQs. LODs were 0.0009–0.0026 µgL^−1^, while LOQs were 0.0027–0.0079 µgL^−1^ [42, 43]. Recovery results at two spiking points (1 and 2 µgL^−1^), each performed in triplicate, evaluate the accuracy and precision. With mean recoveries ranging from 80.11% to 108.46% at 1 µgL^−1^ and 96.54%–117.10% at 2 µgL^−1^, the recovered quantities diligently matched the spiked levels. The slightly elevated recovery percentages observed for specific highly hydrophobic targets such as aldrin (114.66%), heptachlor epoxide (114.80%), *trans*‐chlordane (117.10%), and endrin ketone (116.72%) reflect a minor, well‐documented matrix enhancement phenomenon typical of complex river water matrices in GC–ECD trace analysis. In these scenarios, coextracted natural organic components temporarily occupy active adsorption sites within the injector liner, reducing thermal degradation and allowing a marginally higher mass of these specific analytes to reach the detector. To safeguard data integrity against this potential bias, the analytical workflow incorporated a conservative validation threshold where recoveries up to 120% were deemed acceptable for trace‐level screening, closely aligning with USEPA guidelines for complex environmental samples. The satisfactory precision and reproducibility were verified by relative standard deviations (RSDs), which were typically < 15%. The widely documented validation standard lies 70%–120% for recoveries, with precision (RSD) ≤ 20% [43], slightly higher for certain OCPs; i.e., α‐BHC and 4,4′‐DDT at the higher spiking level could be explained by matrix effects or enhanced detector response, while *cis*‐chlordane and dieldrin have lower (82%–80%) but still within acceptable ranges.

**TABLE 4:** Method validation parameters for the determination of OCPs in Shitalakshya River.

Compounds	Regression equation	RT (min)	*R* ^2^	LOD	LOQ	Recovered amount ± STD		Recovery (%) ± STD	
						For 1 µgL^−1^	For 2 µgL^−1^	For 1 µgL^−1^	For 2 µgL^−1^
α‐BHC	*y* = 101,24*x* + 2831	14.11	0.998	0.0012	0.0037	1.08 ± 0.06	2.15 ± 0.06	108.46 ± 6.50	107.78 ± 3.25
γ‐BHC	*y* = 109,24*x* + 3293	15.68	0.997	0.0018	0.0054	1.05 ± 0.04	1.98 ± 0.07	105.46 ± 4.84	99.01 ± 3.60
β‐BHC	*y* = 7696*x* + 2432	16.06	0.996	0.0023	0.0071	1.05 ± 0.07	1.99 ± 0.14	105.49 ± 7.08	99.53 ± 6.06
δ‐BHC	*y* = 7293*x* + 3468	17.31	0.997	0.0018	0.0054	1.04 ± 0.03	1.97 ± 0.09	104.04 ± 3.08	98.59 ± 4.83
Heptachlor	*y* = 120,50*x* + 3706	17.55	0.995	0.0026	0.0079	0.94 ± 0.07	1.96 ± 0.10	94.69 ± 7.23	98.14 ± 5.24
Aldrin	*y* = 118,71*x* + 3064	18.89	0.997	0.0018	0.0053	1.02 ± 0.04	2.29 ± 0.09	102.70 ± 4.57	114.66 ± 4.75
Heptachlor epoxide	*y* = 133,69*x* + 8663	21.25	0.997	0.0018	0.0053	1.04 ± 0.05	2.29 ± 0.08	104.49 ± 5.38	114.80 ± 4.03
*trans-*Chlordane	*y* = 113,18*x* + 11,649	22.00	0.995	0.0025	0.0076	0.92 ± 0.04	2.34 ± 0.17	92.42 ± 4.99	117.10 ± 8.76
*cis-*Chlordane	*y* = 122,93*x* + 13,649	22.59	0.997	0.0020	0.0061	0.82 ± 0.04	2.23 ± 0.08	82.63 ± 4.94	111.99 ± 4.05
Endosulfan I	*y* = 112,01*x* + 13,321	22.81	0.998	0.0013	0.0041	0.97 ± 0.11	2.32 ± 0.08	97.33 ± 11.51	116.43 ± 4.05
4,4′‐DDE	*y* = 9076*x* + 9667	23.26	0.996	0.0022	0.0068	0.83 ± 0.07	2.15 ± 0.05	83.23 ± 7.09	107.89 ± 2.77
Dieldrin	*y* = 9763*x* + 10,658	23.89	0.997	0.0019	0.0056	0.80 ± 0.04	2.30 ± 0.07	80.11 ± 4.65	115.22 ± 3.75
Endrin	*y* = 6894*x* + 8048	25.07	0.997	0.0020	0.0062	0.88 ± 0.05	2.01 ± 0.08	88.91 ± 5.76	100.67 ± 4.36
4,4′‐DDD	*y* = 5052*x* + 8359	25.50	0.997	0.0019	0.0058	0.86 ± 0.09	2.01 ± 0.06	86.04 ± 9.30	100.64 ± 3.42
Endosulfan II	*y* = 8086*x* + 7814	25.90	0.996	0.0021	0.0065	0.95 ± 0.13	2.28 ± 0.07	95.52 ± 13.45	114.29 ± 3.59
Endrin aldehyde	*y* = 4411*x* + 7274	26.71	0.999	0.0009	0.0027	0.97 ± 0.13	2.15 ± 0.04	97.32 ± 13.02	107.87 ± 2.03
4,4′‐DDT	*y* = 5636*x* + 3962	27.21	0.999	0.0009	0.0027	1.08 ± 0.12	1.93 ± 0.26	108.26 ± 12.90	96.54 ± 13.33
Endosulfan sulfate	*y* = 4953*x* + 8021	28.27	0.996	0.0022	0.0068	0.99 ± 0.11	1.96 ± 0.20	99.65 ± 11.82	98.46 ± 10.28
Methoxychlor	*y* = 2431*x* + 4089	29.34	0.999	0.0010	0.0029	0.94 ± 0.11	2.29 ± 0.27	94.56 ± 11.47	114.65 ± 13.86
Endrin ketone	*y* = 5023*x* + 6432	30.34	0.999	0.0011	0.0035	0.96 ± 0.11	2.33 ± 0.09	96.21 ± 11.37	116.72 ± 4.59

*Note:* The recovery was calculated using 3 replicates in each case.

### 3.2. Spatiotemporal Distribution of OCPs in the Shitalakshya River

**FIGURE 2:**
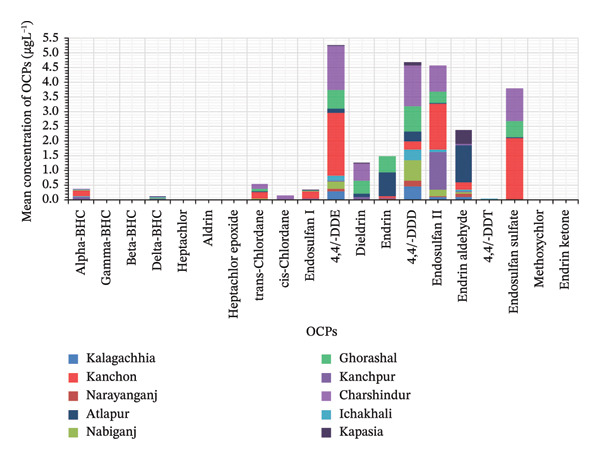
Spatial distribution pattern of OCPs in Shitalakshya river water.

The concentrations of targeted OCPs in the Shitalakshya River exhibited pronounced spatiotemporal variability (Tables 5 and 6). Among the BHC isomers, only α‐BHC and δ‐BHC were consistently detected, reaching maximum concentrations of 0.19 and 0.27 μgL^−1^, respectively, at Kanchon station during L. autumn, while β‐ and γ‐BHC remained BDL throughout the study. These concentrations represented only 19%–27% of the USEPA guideline value (1.0 μgL^−1^) [44], indicating low risk but continued environmental persistence. Chlordane isomers showed heterogeneous distributions, with *trans*‐chlordane peaking at 0.23 μgL^−1^ and *cis*‐chlordane at 0.12 μgL^−1^. Although all values remained below the USEPA guideline (2.0 μgL^−1^) [44], *trans*‐chlordane exceeded the WHO threshold (0.2 μgL^−1^) [45] at specific locations, highlighting localized contamination concerns. The systematic detection of chlordane isomers across the monitoring sites demonstrates that these compounds continue to persist in the aquatic environment despite long‐standing regulatory bans. While their widespread detection confirms environmental persistence and slow degradation rates within the catchment basin, a long‐term temporal trend analysis would be required to determine whether total mass loads are actively increasing or decreasing over consecutive years. Endosulfan residues were also prevalent, with Endosulfan II and endosulfan sulfate reaching maximum concentrations of 1.56 and 2.05 μgL^−1^ at Kanchon and Ghorashal stations, respectively. Despite their occurrence, these levels remained well below the USEPA guideline value (42 μgL^−1^) [44], suggesting limited immediate regulatory concern. In contrast, DDT metabolites were among the most prominent contaminants. Maximum concentrations of 4,4′‐DDE and 4,4′‐DDD reached 2.14 and 4.07 μgL^−1^, respectively, while parent 4,4′‐DDT was detected only at trace levels (< 0.02 μgL^−1^), indicating historical rather than recent inputs. Notably, 4,4′‐DDD exceeded the USEPA guideline value (0.5 μgL^−1^) [44], whereas 4,4′‐DDE slightly surpassed the WHO threshold (2.0 μgL^−1^) [45], suggesting potential environmental and health concerns associated with legacy DDT residues. The extreme concentration spike of hydrophobic analytes such as 4,4′‐DDD and endrin aldehyde specifically during L. autumn 2022 serves as a potential indicator of mass‐driven sediment remobilization combined with a severe contraction of the river’s hydrological dilution capacity. Endrin‐related compounds also exhibited elevated concentrations, with endrin aldehyde and endrin reaching 3.72 and 2.96 μgL^−1^, respectively, while dieldrin peaked at 0.58 μgL^−1^. These concentrations approached or exceeded the corresponding WHO and USEPA guideline values (endrin: 2.0 μgL^−1^; dieldrin: 0.36 μgL^−1^) [44, 45], indicating localized pollution hotspots and potential ecological risks. Methoxychlor and endrin ketone remained BDL throughout the study. Seasonally, most OCPs were detected more frequently and at higher concentrations during the dry seasons (L. autumn, winter, and spring). This pattern can be attributed to the persistence of OCPs in environmental matrices [2], reduced river discharge and dilution capacity during low‐flow periods [46], possible remobilization of contaminated sediments [46], continued release of residues from historical applications and improper disposal [47], and LRAT processes that facilitate their global distribution [48]. Collectively, these factors contribute to the accumulation and enhanced detectability of OCPs during dry seasons in the Shitalakshya River. The spatial distribution of mean OCPs in the Shitalakshya River revealed considerable heterogeneity among sampling stations (Figure 2). Kanchon emerged as the most contaminated site, exhibiting the highest concentrations of α‐BHC (0.19 μgL^−1^), *trans*‐chlordane (0.23 μgL^−1^), Endosulfan I (0.27 μgL^−1^), 4,4′‐DDE (2.14 μgL^−1^), Endosulfan II (1.56 μgL^−1^), and endosulfan sulfate (2.05 μgL^−1^), suggesting potential agricultural and anthropogenic influences. Elevated levels of dieldrin (0.58 μgL^−1^) and 4,4′‐DDD (1.38 μgL^−1^) at Charshindur, together with increased endrin (0.81 μgL^−1^) and endrin aldehyde (1.25 μgL^−1^) at Atlapur, align with localized accumulation of legacy OCP residues. In contrast, several compounds, including γ‐BHC, β‐BHC, heptachlor, aldrin, methoxychlor, and endrin ketone, remained BDL throughout the study area.

**TABLE 5:** Spatiotemporal distribution of OCPs (μgL^−1^) in the Shitalakshya River water.

Name of OCPs	Sampling stations (*n* = 10)										Seasons
	Kalagachhia	Narayanganj	Nabiganj	Kanchpur	Ichakhali	Kanchon	Atlapur	Ghorashal	Charshindur	Kapasia	
α‐BHC	BDL	BDL	BDL	0.10	0.04	0.19	BDL	0.01	0.05	BDL	L. autumn 2022

γ‐BHC	BDL	BDL	BDL	BDL	BDL	BDL	BDL	BDL	BDL	BDL	Whole the year

β‐BHC	BDL	BDL	BDL	BDL	BDL	BDL	BDL	BDL	BDL	BDL	Whole the year

δ‐BHC	0.05	0.01	0.01	0.01	0.01	0.01	BDL	0.01	0.01	BDL	Autumn 2022
	BDL	0.01	BDL	BDL	BDL	0.27	BDL	BDL	BDL	BDL	L. autumn 2022

Heptachlor	BDL	BDL	BDL	BDL	BDL	BDL	BDL	BDL	BDL	BDL	Whole the year
Aldrin	BDL	BDL	BDL	BDL	BDL	BDL	BDL	BDL	BDL	BDL	Whole the year

Heptachlor epoxide	BDL	BDL	BDL	BDL	BDL	BDL	BDL	BDL	BDL	BDL	Whole the year

*trans-*Chlordane	0.02	BDL	0.03	BDL	BDL	0.23	0.01	0.10	0.17	BDL	L. autumn 2022

*cis-*Chlordane	BDL	BDL	BDL	BDL	BDL	BDL	BDL	0.03	0.12	BDL	L. autumn 2022

Endosulfan I	BDL	BDL	0.01	0.01	BDL	BDL	BDL	0.01	BDL	0.03	Autumn 2022
	0.01	BDL	0.02	BDL	BDL	0.27	BDL	BDL	BDL	BDL	L. autumn 2022

4,4′‐DDE	0.31	0.08	0.47	BDL	0.17	2.14	0.12	0.65	1.48	0.03	L. autumn 2022
	BDL	BDL	0.01	0.03	BDL	BDL	BDL	BDL	BDL	BDL	Winter 2023

Dieldrin	BDL	BDL	BDL	0.10	BDL	BDL	0.12	0.45	0.58	0.03	L. autumn 2022

Endrin	0.04	0.01	BDL	0.02	BDL	0.01	BDL	0.01	BDL	BDL	Autumn 2022
	0.01	BDL	0.02	BDL	BDL	0.10	0.81	1.09	0.96	2.96	L. autumn 2022

4,4′‐DDD	1.63	0.69	1.95	BDL	1.05	0.57	1.02	2.51	4.07	0.32	L. autumn 2022
	0.14	0.15	0.06	BDL	0.03	0.19	0.01	0.02	0.05	0.02	Winter 2023
	0.03	0.01	BDL	BDL	BDL	BDL	BDL	BDL	BDL	BDL	Spring 2023
	0.01	0.02	0.01	0.01	0.02	0.01	0.03	0.01	0.01	0.03	Summer 2023

Endosulfan II	0.11	0.03	0.21	1.29	0.07	1.56	0.02	0.40	0.88	BDL	L. autumn 2022

Endrin aldehyde	BDL	BDL	BDL	BDL	BDL	BDL	3.72	BDL	BDL	1.34	L. autumn 2022
	0.16	0.21	0.06	0.05	0.03	0.45	0.01	0.02	0.07	0.03	Winter 2023
	0.02	0.04	0.03	0.02	0.03	0.04	0.03	0.02	0.02	0.05	Summer 2023

4,4′‐DDT	BDL	BDL	BDL	0.01	0.01	BDL	BDL	BDL	BDL	BDL	Autumn 2022
	BDL	BDL	0.02	BDL	BDL	BDL	BDL	BDL	BDL	BDL	L. autumn 2022

Endosulfan sulfate	BDL	0.04	BDL	BDL	BDL	2.05	0.03	0.56	1.12	BDL	L. autumn 2022

Methoxychlor	BDL	BDL	BDL	BDL	BDL	BDL	BDL	BDL	BDL	BDL	Whole the year

Endrin ketone	BDL	BDL	BDL	BDL	BDL	BDL	BDL	BDL	BDL	BDL	Whole the year

**TABLE 6:** A standard framework of OCPs (μgL^−1^) in the Shitalakshya River water along with their standard guideline limits.

Name of OCPs	Conc. ranges	Detection frequencies, DF (%)	Max. conc.	USEPA guideline limit [44]	WHO guideline limit [45]	Compliance status	Exceedance seasons	Exceedance locations
α‐BHC	BDL‐0.19	8.33	0.19	1.00	—	Below guideline	None	None

γ‐BHC	BDL	0.00	—	0.20	2.00	Below guideline	None	None

β‐BHC	BDL	0.00	—	1.00	0.30	Below guideline	None	None

δ‐BHC	BDL‐0.05	13.34	0.27	1.00	—	Below guideline	None	None
	BDL‐0.27	3.34						

Heptachlor	BDL	0.00	—	0.52	0.03	Below guideline	None	None

Aldrin	BDL	0.00	—	0.36	0.03	Below guideline	None	None

Heptachlor epoxide	BDL	0.00	—	0.20	0.20	Below guideline	None	None

*trans-*Chlordane	BDL‐0.23	10.00	0.23	2.00	0.20	Exceeded WHO; below USPA	L. autumn 2022	Kanchon

*cis-*Chlordane	BDL‐0.12	3.34	0.12	2.00	0.20	Below guideline	None	None

Endosulfan I	BDL‐0.03	6.67	0.27	42.00	—	Below guideline	None	None
	BDL‐0.27	5.00						

4,4′‐DDE	BDL‐2.14	15.00	2.14	—	2.00	Exceeded WHO	L. autumn 2022	Kanchon
	BDL‐0.03	3.34						

Dieldrin	BDL‐0.58	8.33	0.58	0.36	0.03	Exceeded guideline	L. autumn 2022	Charshindur

Endrin	BDL‐0.04	8.33	2.96	2.00	0.30	Exceeded guideline	L. autumn 2022	Kapasia
	BDL‐2.96	11.67						

4,4′‐DDD	BDL‐4.07	15.00	4.07	0.50	—	Exceeded USEPA	L. autumn 2022	Charshindur
	BDL‐0.19	15.00						
	BDL‐0.03	3.34						
	0.01–0.03	16.67						

Endosulfan II	BDL‐1.56	15.00	1.56	42.0	—	Below guideline	None	None

Endrin aldehyde	BDL‐3.72	3.34	3.72	0.10	—	Exceeded USEPA	L. autumn 2022	Atlapur
	0.01–0.45	16.67						
	0.02–0.05	16.67						

4,4′‐DDT	BDL‐0.01	3.34	0.02	0.50	2.00	Below guideline	None	None
	BDL‐0.02	1.67						

Endosulfan sulfate	BDL‐2.05	8.33	2.05	42.0	—	Below guideline	None	None

Methoxychlor	BDL	0.00	—	42.0	20.00	Below guideline	None	None

Endrin ketone	BDL	0.00	—	—	—	Below guideline	None	None

**FIGURE 3:**
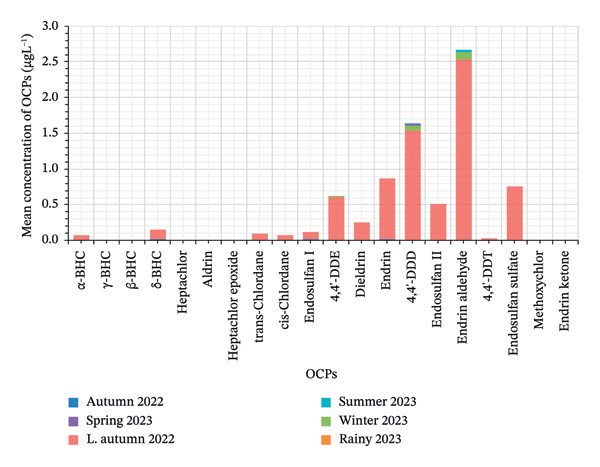
Seasonal distribution pattern of OCPs in Shitalakshya river water.

The monitoring of OCPs in the Shitalakshya River revealed a distinct seasonal contamination gradient, characterized by a massive peak during L. autumn 2022 (Figure 3). During this period, 13 out of 20 assessed OCP compounds were detected. Endrin aldehyde exhibited the highest individual concentration (2.530 μgL^−1^), followed closely by 4,4′‐DDD (1.534 μgL^−1^) and endrin (0.850 μgL^−1^). In contrast, OCP residues dramatically declined in subsequent seasons; only minor traces of 4,4′‐DDD and endrin aldehyde persisted into winter, spring, and summer 2023, while concentrations fell entirely BDL by the rainy 2023 season. This stark temporal variation may be driven by hydrological fluctuations and local agricultural cycles. The highly elevated pollution profile in L. autumn aligns with the low‐flow period of the Shitalakshya River, where reduced water volume minimizes dilution capabilities, concentrating runoff from surrounding agricultural fields. Conversely, the complete absence of detectable OCPs during the rainy season is attributed to the massive dilution effect of monsoon precipitation and elevated river discharge. The persistent presence of DDT metabolites (4,4′‐DDE and 4,4′‐DDD) over parent 4,4′‐DDT suggests historical usage and ongoing degradation within the aquatic ecosystem.

### 3.3. Human Health Risk Assessment for the Exposure of OCPs

The majority of OCPs possess hydrophobic characteristics, which cause them to stick strongly to suspended particles and debris in water bodies. These OCPs can separate and be discharged into the water column, especially when there is water turbulence, which poses serious dangers to aquatic life and public health [49].

The findings from the evaluation of cancer risk (*C*_*r*_), as suggested by *P*, *R*, and *H*_*q*_, can be observed in Table 7. For the vast majority of targeted OCPs, the calculated *C*_*r*_, *P*, *R*, and *H*_*q*_ values fell safely below standard regulatory risk thresholds, indicating acceptable baselines for both children and adults across most sampling periods [36, 38]. However, a critical exception was observed for 4,4′‐DDD during L. autumn 2022, where the *H*_*q*_ surpassed the risk threshold of 1.0 across all age groups (Table 7), driven by elevated concentrations (*A* = 1.4 × 10^−3^). This suggests a definitive localized noncarcinogenic health hazard during the low‐flow dry period. Therefore, acute carcinogenic and noncarcinogenic risks are minimal for most compounds with the exception of 4,4′‐DDD during L. autumn, representing an active health concern that demands targeted mitigation [37]. To account for the simultaneous exposure to multiple contaminants, the cumulative health risk of the detected OCPs was evaluated (Table 7). The cumulative HI, which represents the sum of individual *H*_*q*_, reached 4.3 × 10^0^ (4.3) for children aged 0–6 years, 4.7 × 10^0^ (4.7) for adolescents aged 7–17 years, and 5.1 × 10^0^ (5.1) for adults. Because an HI > 1.0 suggests a potential for adverse noncarcinogenic health effects, these findings reveal that cumulative exposure poses a significant health risk to the local population relying on the Shitalakshya River water, even though most individual OCPs fell below the threshold. The primary contributors to this noncarcinogenic burden were 4,4′‐DDD, 4,4′‐DDE, and endosulfan compounds, particularly during the late autumn 2022 period. Conversely, the total cumulative lifetime cancer risk (*R* and *C*_*r*_) values remained within or slightly below the acceptable USEPA safety range (10^−6^ to 10^−4^), indicating that noncarcinogenic chronic toxicity is the primary health concern for this aquatic system.

**TABLE 7:** Human health risk potential data of OCPs in Shitalakshya River.

OCPs	Risk assessment parameters											Season/year
	*A* ^∗^ (mgL^−1^)	P_(0–6 years)_	P_7–17 years_	P_adult_	*H*_*q*__(0–6 years)_	*H*_*q*__(7–17 years)_	*H*_*q*__(adult)_	R_0–6 years_	R_7–17 years_	R_adult_	*C*_*r*_	
Alpha‐BHC	3.9 × 10^−5^	7.6 × 10^−7^	8.3 × 10^−7^	8.9 × 10^−7^	3.8 × 10^−2^	4.2 × 10^−2^	4.5 × 10^−2^	6.6 × 10^−8^	1.3 × 10^−7^	3.8 × 10^−7^	4.6 × 10^−14^	L. autumn 2022

Delta‐BHC	1.2 × 10^−5^	2.4 × 10^−7^	2.6 × 10^−7^	2.7 × 10^−7^	1.2 × 10^−2^	1.3 × 10^−2^	1.4 × 10^−2^	2.0 × 10^−8^	4.0 × 10^−8^	1.2 × 10^−7^	1.4 × 10^−14^	Autumn 2022
	2.8 × 10^−5^	5.5 × 10^−7^	6.0 × 10^−7^	6.4 × 10^−7^	2.7 × 10^−2^	3.0 × 10^−2^	3.2 × 10^−2^	4.7 × 10^−8^	9.4 × 10^−8^	2.7 × 10^−7^	3.3 × 10^−14^	L. autumn 2022

*trans-*Chlordane	5.6 × 10^−5^	1.1 × 10^−6^	1.2 × 10^−6^	1.3 × 10^−6^	5.5 × 10^−2^	6.0 × 10^−2^	6.4 × 10^−2^	9.4 × 10^−8^	1.9 × 10^−7^	5.5 × 10^−7^	6.5 × 10^−14^	L autumn 2022

*cis-*Chlordane	1.5 × 10^−5^	2.9 × 10^−7^	3.2 × 10^−7^	3.4 × 10^−7^	1.5 × 10^−2^	1.6 × 10^−2^	1.7 × 10^−2^	2.5 × 10^−8^	5.0 × 10^−8^	1.5 × 10^−7^	1.8 × 10^−14^	L. autumn 2022

Endosulfan I	6.0 × 10^−6^	1.2 × 10^−7^	1.3 × 10^−7^	1.4 × 10^−7^	5.9 × 10^−3^	6.4 × 10^−3^	6.9 × 10^−3^	1.0 × 10^−8^	2.0 × 10^−8^	5.9 × 10^−8^	7.0 × 10^−15^	Autumn 2022
	3.0 × 10^−5^	5.9 × 10^−7^	6.4 × 10^−7^	6.9 × 10^−7^	2.9 × 10^−2^	3.2 × 10^−2^	3.4 × 10^−2^	5.0 × 10^−8^	1.0 × 10^−7^	2.9 × 10^−7^	3.5 × 10^−14^	L. autumn 2022

4,4′‐DDE	5.5 × 10^−4^	1.1 × 10^−5^	1.2 × 10^−5^	1.2 × 10^−5^	5.3 × 10^−1^	5.8 × 10^−1^	6.2 × 10^−1^	9.2 × 10^−7^	1.8 × 10^−6^	5.3 × 10^−6^	6.4 × 10^−13^	L. autumn 2022
	4.0 × 10^−6^	7.8 × 10^−8^	8.5 × 10^−8^	9.1 × 10^−8^	3.9 × 10^−3^	4.3 × 10^−3^	4.6 × 10^−3^	6.7 × 10^−9^	1.3 × 10^−8^	3.9 × 10^−8^	4.7 × 10^−15^	Winter 2023

Dieldrin	1.3 × 10^−4^	2.5 × 10^−6^	2.7 × 10^−6^	2.9 × 10^−6^	1.3 × 10^−1^	1.4 × 10^−1^	1.5 × 10^−1^	2.2 × 10^−7^	4.3 × 10^−7^	1.3 × 10^−6^	1.5 × 10^−13^	L. autumn 2022

Endrin	9.0 × 10^−6^	1.8 × 10^−7^	1.9 × 10^−7^	2.1 × 10^−7^	8.8 × 10^−3^	9.6 × 10^−3^	1.0 × 10^−2^	1.5 × 10^−8^	3.0 × 10^−8^	8.8 × 10^−8^	1.1 × 10^−14^	Autumn 2022
	6.0 × 10^−4^	1.2 × 10^−5^	1.3 × 10^−5^	1.4 × 10^−5^	5.8 × 10^−1^	6.3 × 10^−1^	6.8 × 10^−1^	1.0 × 10^−6^	2.0 × 10^−6^	5.8 × 10^−6^	6.9 × 10^−13^	L. autumn 2022

4,4′‐DDD	1.4 × 10^−3^	2.7 × 10^−5^	2.9 × 10^−5^	3.2 × 10^−5^	1.4 × 10^0^	1.5 × 10^0^	1.6 × 10^0^	2.3 × 10^−6^	4.6 × 10^−6^	1.4 × 10^−5^	1.6 × 10^−12^	L. autumn 2022
	6.7 × 10^−5^	1.3 × 10^−6^	1.4 × 10^−6^	1.5 × 10^−6^	6.6 × 10^−2^	7.1 × 10^−2^	7.7 × 10^−2^	1.1 × 10^−7^	2.2 × 10^−7^	6.6 × 10^−7^	7.8 × 10^−14^	Winter 2023
	4.0 × 10^−6^	7.8 × 10^−8^	8.5 × 10^−8^	9.1 × 10^−8^	3.9 × 10^−3^	4.3 × 10^−3^	4.6 × 10^−3^	6.7 × 10^−9^	1.3 × 10^−8^	3.9 × 10^−8^	4.7 × 10^−15^	Spring 2023
	1.6 × 10^−5^	3.1 × 10^−7^	3.4 × 10^−7^	3.7 × 10^−7^	1.6 × 10^−2^	1.7 × 10^−2^	1.8 × 10^−2^	2.7 × 10^−8^	5.4 × 10^−8^	1.6 × 10^−7^	1.9 × 10^−14^	Summer 2023

Endosulfan II	4.6 × 10^−4^	9.0 × 10^−6^	9.7 × 10^−6^	1.0 × 10^−5^	4.5 × 10^−1^	4.9 × 10^−1^	5.2 × 10^−1^	7.7 × 10^−7^	1.5 × 10^−6^	4.5 × 10^−6^	5.3 × 10^−13^	L. autumn 2022

Endrin aldehyde	5.1 × 10^−4^	9.9 × 10^−6^	1.1 × 10^−5^	1.2 × 10^−5^	5.0 × 10^−1^	5.4 × 10^−1^	5.8 × 10^−1^	8.5 × 10^−7^	1.7 × 10^−6^	5.0 × 10^−6^	5.9 × 10^−13^	L. autumn 2022
	1.1 × 10^−4^	2.1 × 10^−6^	2.3 × 10^−6^	2.5 × 10^−6^	1.1 × 10^−1^	1.2 × 10^−1^	1.2 × 10^−1^	1.8 × 10^−7^	3.6 × 10^−7^	1.1 × 10^−6^	1.3 × 10^−13^	Winter 2023
	3.0 × 10^−5^	5.9 × 10^−7^	6.4 × 10^−7^	6.9 × 10^−7^	2.9 × 10^−2^	3.2 × 10^−2^	3.4 × 10^−2^	5.0 × 10^−8^	1.0 × 10^−7^	2.9 × 10^−7^	3.5 × 10^−14^	Summer 2023

4,4′‐DDT	2.0 × 10^−6^	3.9 × 10^−8^	4.3 × 10^−8^	4.6 × 10^−8^	2.0 × 10^−3^	2.1 × 10^−3^	2.3 × 10^−3^	3.4 × 10^−9^	6.7 × 10^−9^	2.0 × 10^−8^	2.3 × 10^−15^	Autumn 2022
	2.0 × 10^−6^	3.9 × 10^−8^	4.3 × 10^−8^	4.6 × 10^−8^	2.0 × 10^−3^	2.1 × 10^−3^	2.3 × 10^−3^	3.4 × 10^−9^	6.7 × 10^−9^	2.0 × 10^−7^	2.3 × 10^−15^	L. autumn 2022

Endosulfan sulfate	3.8 × 10^−4^	7.4 × 10^−6^	8.1 × 10^−6^	8.7 × 10^−6^	3.7 × 10^−1^	4.0 × 10^−1^	4.3 × 10^−1^	6.4 × 10^−7^	1.3 × 10^−6^	3.7 × 10^−6^	4.4 × 10^−13^	L. autumn 2022

Cumulative risk		8.7 × 10^−5^	9.4 × 10^−5^	1.0 × 10^−4^	4.3 × 10^0^	4.7 × 10^0^	5.1 × 10^0^	7.4 × 10^−6^	1.5 × 10^−5^	4.3 × 10^−5^	5.2 × 10^−12^	

*Note:* *A* ^∗^ is the average value of OCP amounts.

### 3.4. Principal Component Analysis of OCPs for Spatial Dataset

**FIGURE 4:**
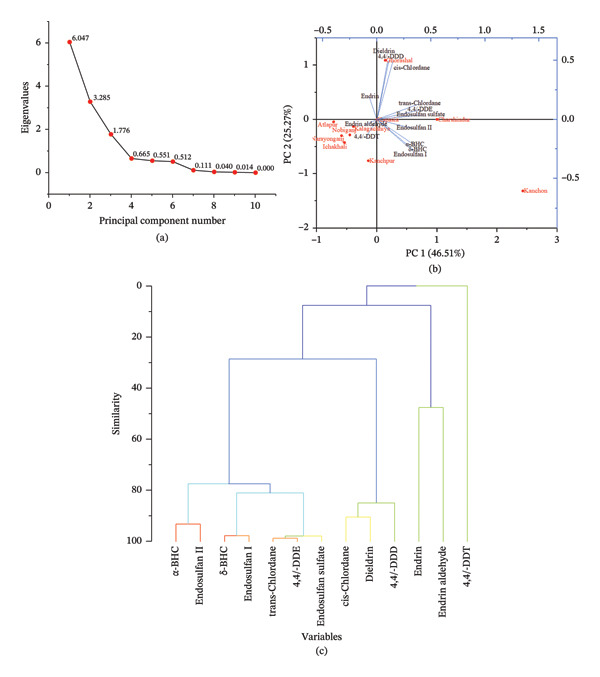
Scree plot (a), biplot of PCA (b), and cluster analysis (c) of the spatial dataset of OCPs.

The scree plot (Figure 4a) for OCPs shows that the first two PCs have high eigenvalues (6.047 and 3.285), indicating that they capture the main variance in the spatial dataset. The clear elbow after the second component indicates that these two PCs are enough to clarify the primary spatial distribution patterns of OCP pollution. PCA was used as an exploratory multivariate tool to identify potential patterns and associations among variables. The interpretation of PCA results was carried out in a qualitative manner to understand grouping behavior and possible influencing factors, rather than for quantitative source apportionment. The PCA of OCP contamination in Shitalakshya River wastewater is shown in Figure 4b. The first two principal components, PC1 (46.51%) and PC2 (25.27%), cumulatively clarify 71.78% of the total variance. PC1 is positively interrelated with Endosulfan I, Endosulfan II, endosulfan sulfate, α‐BHC, δ‐BHC, 4,4′‐DDE, and *trans-*Chlordane. Kanchan station has a strong correlation with these OCPs, implying their prevalence. Conversely, Atlapur, Nabiganj, Narayanganj, Ichakhali, and Kalagachhia clustered toward lower pollution of these OCPs. PC2 represented the effect of Dieldrin, 4,4′‐DDD, *cis*‐Chlordane, and endrin. Ghorashal showed high levels of these PC2‐related OCPs. Kanchpur’s position implies a distinct OCP pattern not controlled by PC2 variables. The determined distinct OCP profiles, such as the dominance of endosulfans and BHCs in Kanchan and dieldrin and chlordane in Ghorashal, reflect site‐specific pollution patterns. While these statistical groupings are highly illustrative, they serve as indicators of co‐occurrence rather than definitive proof of active physical sources. This variation may tentatively be attributed to historical, nonpoint source agricultural runoff or legacy residues embedded within surrounding urban catchment spaces; however, tracking exact point‐source industrial discharges or illegal modern applications remains outside the scope of exploratory multivariate modeling. To systematically differentiate whether the observed OCP contamination stems from active, modern applications or from the weathering of historical inputs, pesticide diagnostic ratios (4,4′‐DDT/(4,4′‐DDE + 4,4′‐DDD)) were calculated. Diagnostic ratios across all seasons approach zero (<< 0.5), providing empirical proof that contamination may stem entirely from weathered, legacy agricultural burdens rather than active modern inputs. The distribution of HCH isomers was analyzed to differentiate between historical technical HCH inputs and possible lindane use. α‐BHC (0.01–0.19 μgL^−1^) and δ‐BHC (0.01–0.27 μgL^−1^) were detected at trace levels, while γ‐BHC (lindane) was mostly below detection limits across sampling periods. The consistently high *α*/*γ* ratios (> 7), exceeding the typical technical HCH range (3–7), are consistent with no evidence of recent lindane application. The co‐occurrence of stable β‐ and δ‐HCH, along with isomer transformation patterns, confirms that contamination in the Shitalakshya River reflects only legacy residues from historical technical HCH use.

The dendrogram (Figure 4c) evidently groups the pesticides depending on their similarity in river water. A distinct cluster was formed by α‐BHC, Endosulfan II, δ‐BHC, Endosulfan I, *trans*‐chlordane, 4,4′‐DDE, and endosulfan sulfate, indicating a high degree of similarity among these compounds. This suggests mutual environmental incidences or diffuse sources for the OCPs, including BHC, endosulfan, and a DDT metabolite. Another discrete group clustering together includes *cis*‐chlordane, dieldrin, and 4,4′‐DDD, suggesting their related occurrence or nature. Notably, endrin and its degradation product, i.e., endrin aldehyde, make a constricted cluster, ensuring their direct correlation. Conversely, 4,4′‐DDT seems to be quite distinct from most other OCPs, connecting the overall group at a very low similarity level. This could suggest different major sources or a unique degradation nature compared to other OCPs. These clustering patterns help identify groups of pesticides that may originate from similar anthropogenic activities or undergo comparable transport and transformation processes in the river ecosystem.

### 3.5. Comparative studies of OCPs Among the Peripheral Rivers of Dhaka

The quantities of OCPs in Dhaka city’s peripheral river waters are compared in Table 8. The results of the current analysis for the Shitalakshya River are compared to those of a different study carried out in 2021 by [50]. For the Shitalakshya River in this study and other rivers [50], the concentrations of certain OCPs, such as gamma‐BHC, beta‐BHC, heptachlor, aldrin, heptachlor epoxide, cis‐chlordane, dieldrin, endrin aldehyde, methoxychlor, and endrin ketone, are listed as BDL. Endosulfan I, 4,4′‐DDE, endrin, 4,4′‐DDD, 4,4′‐DDT, and endosulfan sulfate concentrations are reported for the Shitalakshya River in this study as well as the other rivers. It provides information about possible fluctuations in OCP contamination levels by displaying the presence or absence under investigation. Another study in Bangladesh for the surveillance of OCP residues by GC–MS analysis in surface water samples from agricultural areas reported that 4,4′‐DDT, heptachlor, and lindane were present in trace amounts in a small number of water samples from irrigated areas, but the majority of the samples were found to be free of contamination. Aldrin, dieldrin, and endrin were not found in any measurable amounts in any of the agricultural field samples under investigation [51]. To monitor the existence of OCPs in pond water, farmed land, and tube‐well water, 10 administrative divisions in the Dhamrai subdistrict of the Dhaka division, Bangladesh, were the subject of an extra study project [21]. There were no indications of contamination from the 4,4′‐DDT, derivatives 4,4′‐DDE, and 4,4′‐DDD in any of the tested water samples.

**TABLE 8:** Comparative studies of OCPs (μgL^−1^) in the peripheral river waters of Dhaka.

OCPs	Shitalakshya (present study)	Other study [50]				
		Shitalakshya	Dhaleshwari	Buriganga	Turag	Balu
Alpha‐BHC	0.039	NS	NS	NS	NS	NS
Gamma‐BHC	BDL	BDL	BDL	BDL	BDL	BDL
Beta‐BHC	BDL	NS	NS	NS	NS	NS
Delta‐BHC	0.200	NS	NS	NS	NS	NS
Heptachlor	BDL	BDL	BDL	BDL	BDL	BDL
Aldrin	BDL	0.482	0.241	0.834	0.453	0.235
Heptachlor epoxide	BDL	0.220	0.098	0.321	0.231	0.165
*trans-*Chlordane	0.056	BDL	BDL	BDL	BDL	BDL
*cis-*Chlordane	0.015	BDL	BDL	BDL	BDL	BDL
Endosulfan I	0.018	0.094	0.074	0.123	BDL	0.078
4,4′‐DDE	0.274	0.882	0.832	1.782	1.267	1.023
Dieldrin	0.128	NS	NS	NS	NS	NS
Endrin	0.302	0.776	0.997	1.120	1.090	0.989
4,4′‐DDD	0.367	0.856	BDL	1.421	1.236	1.007
Endosulfan II	0.457	BDL	BDL	0.242	0.175	0.080
Endrin aldehyde	0.215	NS	NS	NS	NS	NS
4,4′‐DDT	0.002	0.605	0.993	1.243	1.076	1.243
Endosulfan sulfate	0.380	BDL	BDL	0.023	BDL	BDL
Methoxychlor	BDL	BDL	BDL	BDL	BDL	BDL
Endrin ketone	BDL	0.098	0.074	0.273	0.123	0.032

*Note:* The values reported are all mean values.

Abbreviations: BDL, below detection limit; NS, not studied.

A follow‐up study was conducted to monitor the residue of DDT or its derivatives in tube‐well water, paddy field water, and pond water that were gathered from 10 administrative divisions in Nagarpur Upazila, which is in the Tangail district. In the same way, water from nine administrative divisions in Saturia Upazila, which is located in the Bangladeshi district of Manikganj, was gathered together with water from the Dhaleshwari and Gazikhali rivers for use in paddy fields [52]. No evidence of pollution by 4,4′‐DDT or its derivatives, 4,4′‐DDE and 4,4′‐DDD, was found in any of the tested water samples. However, further research is required in different matrices like soil, fish, or any other bioindicator to address the critical points highlighted above, enabling a more comprehensive understanding of the environmental, health, and ecological implications of OCP contamination and informing effective pollution management strategies.

#### 3.5.1. Spatial and Temporal Comparative Dynamics

The comparative analysis presented in Table 8 reveals distinct shifts in the OCP profiles of the Shitalakshya River when contrasted with historical regional data [50]. These variations are not arbitrary but reflect an intersection of shifting pesticide regulatory compliance, environmental half‐lives, and hydrological dynamics: (i) Markedly Reduced DDT and Endrin Concentrations: A key finding in Table 8 is the substantial decline in active DDT isomers and endrin levels compared to previous studies. This reduction is highly indicative of the long‐term efficacy of the Stockholm Convention bans and stricter regional regulatory enforcement over the past decade. Because primary applications have ceased, the residual concentrations observed in this study represent highly weathered, legacy baselines. Furthermore, compounds like endrin degrade relatively faster in warm, biologically active tropical aquatic environments via photolysis and microbial action, accelerating their removal from the water column when modern inputs are absent. (ii) Comparatively Elevated Endosulfan II Levels: Endosulfan II levels appear comparatively elevated or persistent relative to older historical data. This trend aligns with the timeline of regulatory bans; endosulfan was placed on the Stockholm Convention restricted list much later (2011) than primary legacy OCPs like DDT and aldrin (2004) [53]. Because of its more recent widespread use in regional agriculture, a larger reservoir of endosulfan residues remains bound to catchment soils. Under intense monsoon and postmonsoon hydrological runoff conditions, these relatively “younger” legacy residues are continuously transported into the river system, maintaining elevated concentrations in the water column compared to the older, more depleted legacy OCP pools. (iii) Analytical Methodology Considerations: Beyond environmental factors, variations in analytical methodologies between studies must be considered. While older historical assessments often relied on LLE–GC–MS or other conventional detection approaches, this study utilized a highly optimized LLE–GC–ECD. The high sensitivity and specificity of the ECD specifically toward halogenated compounds allow for the precise quantification of trace‐level OCP metabolites, which may account for differences in detection limits and reported baseline concentrations between monitoring eras.

### 3.6. Study Limitations and Uncertainty

Samples were collected in amber HDPE bottles, which were selected over amber glass due to logistical considerations and reduced risk of breakage during field transport. Amber glass is generally preferred for OCP analysis because of the hydrophobic nature of these compounds and their potential adsorption onto polymer surfaces [54]. To minimize potential analyte loss, samples were extracted within 48 h of collection [55]. Validation through spiked recovery experiments under simulated storage conditions yielded recovery rates of 80.11%–117.10%. These results confirm that the adopted storage and extraction protocol effectively minimized analyte adsorption and maintained sample integrity throughout the analytical process [54]. The reliance on single, discrete grab samples per campaign, without field duplicates or composite blending, may miss short‐term, localized chemical fluctuations. However, this approach was chosen to preserve real‐time point‐source signatures, with analytical robustness maintained through rigorous triplicate laboratory injections and matrix spike controls. Another key limitation of this study is that the reported concentrations (Table 5) were derived from single measurements of individual water samples, preventing the quantification of analytical variability through replicate analyses. In addition, the seasonal mean values (Table 7) were computed across different sampling periods rather than replicate measurements of identical samples, limiting the ability to assess formal uncertainty and variability. As a result, inferential statistical approaches such as ANOVA could not be appropriately applied, since the assumptions required for robust hypothesis testing were not fully satisfied. Therefore, the present work primarily relies on descriptive statistics, spatial–temporal patterns, and multivariate analyses to interpret the data. Future studies should incorporate replicate sampling, repeated analyses, and probabilistic risk assessment frameworks to more comprehensively capture analytical uncertainty and to enable rigorous statistical evaluation of seasonal and spatial differences. Several factors contribute to the uncertainty in this risk assessment. First, the use of point‐in‐time sampling may not fully capture the temporal variability of OCP concentrations, particularly during extreme seasonal events. Second, while our recovery experiments confirm the suitability of our storage and extraction methods, inherent uncertainties in the toxicological reference values used in the risk calculations can impact the final risk estimates. Furthermore, the assumption of additive toxicity for HI does not account for potential synergistic or antagonistic effects among the different compounds analyzed.

### 3.7. Recommendation and Implication

Although the human health risk assessment suggests that the majority of targeted OCPs generally remain within acceptable regulatory limits, the persistent baseline presence of these legacy chemicals and the targeted seasonal threshold breach of 4,4′‐DDD (*H*_*q*_ > 1) during L. autumn highlight critical focal points for local water resource management. To mitigate these specific issues, targeted interventions should replace blanket regional bans. First, environmental enforcement must focus on strict seasonal monitoring during low‐flow periods (autumn and L. autumn), when reduced dilution capacity exacerbates contaminant concentrations. Second, since legacy OCP metabolites likely reside within riverbed substrates, future dredging or infrastructural developments along the Shitalakshya River must be carefully regulated to minimize sediment remobilization. Finally, agricultural extension services should continue educating farming communities along the river basin regarding sustainable, nonpersistent pesticide alternatives to ensure that legacy contaminant levels continue their downward trajectory rather than experiencing modern inputs. To maximize remediation effectiveness, management efforts should focus on the identified contamination hotspots of Kanchon, Ghorashal, and Charshindur. Localized activated carbon filtration systems are recommended for Ghorashal and Charshindur, where elevated levels of persistent hydrophobic OCP metabolites such as 4,4′‐DDD and dieldrin were detected, while constructed wetlands may help reduce inputs of endosulfans and BHCs associated with agricultural and urban runoff entering the river near Kanchon. In addition, regulatory authorities should prioritize monitoring and enforcement activities within these hotspot zones rather than applying uniform basin‐wide measures. Particular attention should be given to low‐flow seasons, when reduced dilution may enhance contaminant accumulation and associated environmental risks.

## 4. Conclusion

This study illustrated the spatiotemporal distribution, ecological and human health risks, and potential sources of 20 OCPs in the surface water of the Shitalakshya River using a validated LLE–GC–ECD method. The method has excellent sensitivity, linearity, and recovery, confirming reliable quantification. The analytical results confirm that specific legacy OCPs endure in the water column despite long‐standing regulatory bans, with the highest total concentrations and detection frequencies occurring during the low‐flow dry periods (L. autumn and autumn), and moderately in summer and spring. Several OCPs, including 4,4′‐DDE, dieldrin, endrin, and 4,4′‐DDD, exceeded threshold levels at specific stations, suggesting localized accumulation linked to urbanized and agriculturally influenced river stretches. Multivariate analyses suggest mixed contributions from historical residues and contemporary land‐use activities, although these patterns remain indicative rather than confirmatory of active sources. Health risk assessment shows that both carcinogenic and noncarcinogenic risks are generally within acceptable limits across most seasons, suggesting limited immediate risk from direct water ingestion. However, a seasonal exception was observed for 4,4′‐DDD during L. autumn, where *H*_*q*_ > 1, indicating potential noncarcinogenic concern. Overall, despite low current exposure risks, the persistence and bioaccumulative nature of these legacy pollutants highlight a chronic environmental issue, underscoring the need for stronger regulation, improved waste management, and continuous seasonal monitoring.

## Author Contributions

Md. Mazharul Islam: writing–review and editing, writing–original draft, methodology, investigation, formal analysis, data curation, and conceptualization.

Mohammad Shoeb: writing–original draft, methodology, investigation, data curation, conceptualization, resources, and funding acquisition.

Md. Iqbal Rouf Mamun: writing–original draft, methodology, investigation, and data curation.

## Funding

This research was funded by the Swedish Research Council (Vetenskapsrådet), 2021‐04909, under the project “Antibiotic Resistance: The Role of Chemical Pollutants in Urban Wastewater.”

## Conflicts of Interest

The authors declare no conflicts of interest.

## Data Availability

The data supporting the findings of this study are available within the article.
